# Prevalence and characterization of hepatitis B and C virus infections in a needle-sharing population in Northern China

**DOI:** 10.1186/s12889-015-1808-0

**Published:** 2015-05-02

**Authors:** Cheng-Jun Xu, Cui-Ping Zhang, Bi-Fen Luo, Li-Jun Liu, Yun-Zhong Wang, Xiao-Hong Wang, Qiu-Jie He, Shan-Shan Zhou, Wei-Shan Guo, Jiu-Heng Wang, Rui-Feng Yang, Hai-Ying Zhang, Hui-Ying Rao, Bo Feng, Lai Wei

**Affiliations:** Department of Infectious Diseases, People’s Hospital of Kuancheng Manchu Nationality Autonomous County, Hebei, 067600 China; Peking University People’s Hospital, Peking University Hepatology Institute, Beijing Key Laboratory of Hepatitis C and Immunotherapy for Liver Diseases, No. 11 Xizhimen South Street, Beijing, 100044 China

**Keywords:** Prevalence, Hepatitis C virus, Hepatitis B virus, Hepatitis, Intravenous drug user

## Abstract

**Background:**

The epidemiologies of hepatitis C virus (HCV) and hepatitis B virus (HBV) infections in specific populations in certain areas of China are poorly understood. A pilot survey of HCV/HBV infections was carried out in villages in Kuancheng County, Heben Province, where injection of sodium benzoate or amphetamines using shared needles has been a common practice. The aims of this study were to analyze the endemicity and characterize HCV/HBV infections in this population.

**Methods:**

Data on demographic characteristics and drug abuse were collected from individuals who signed informed consent forms. Serum HCV antibody (anti-HCV), hepatitis B surface antigen (HBsAg), and hepatitis B core antibody (anti-HBc) were measured in all participants. HCV RNA was measured in samples positive for anti-HCV using real-time polymerase chain reaction.

**Results:**

Among 852 participants from 11 villages, 49.9% had used sodium benzoate or amphetamine at least once, by intravenous injection. The overall prevalence of anti-HCV, HCV RNA, anti-HBc, HBsAg, and HCV/HBV co-infection was 37.1%, 26.6%, 67.7%, 10.7%, and 30.0%, respectively. Two-hundred-twenty-three of 227 (98.2%) participants positive for HCV RNA were aged >40 years. Co-infection was related to sex, age, number of injections, and time from first injection. The rate of spontaneous HCV RNA clearance was 28.2% (89/316), and was related to the number of injections, time from first injection, and HBsAg positivity. However, HBsAg was related to the anti-HBc signal/cut-off ratio rather than to the above parameters. Trend tests demonstrated that the prevalence of anti-HCV, HCV RNA, and anti-HBc was related to the number of injections (*P* < 0.001), while HBsAg prevalence was not (*P* = 0.347).

**Conclusions:**

The prevalence of HCV and HBV infection is likely to be high among individuals older than 40 years in areas of needle sharing, and one-time screening for HCV infection should be offered to these populations.

## Background

The World Health Organization has estimated that around 150 million and >240 million people worldwide are chronically infected with hepatitis C virus (HCV) and hepatitis B virus (HBV), respectively, which are responsible for almost 1 million deaths every year [1,2]. Chronic HCV and HBV infections are the most important causes of end-stage liver diseases, including decompensated cirrhosis and hepatocellular carcinoma [3]. Their early detection, diagnosis, and treatment is the key to inhibiting or even eradicating both these viruses. However, it is important to understand the epidemiologies of HCV and HBV infections to allow the introduction of effective screening programs.

Viral hepatitis is a major public health problem in China. A nationwide cross-sectional seroepidemiological study of hepatitis virus infections in 1992 showed an overall prevalence of anti-HCV antibody of 3.2% (range 0.9–5.1%) among 66,975 subjects from 30 provinces [4]. Another seroepidemiological study using blood samples and data from a nationwide survey of hepatitis B in 2006 found an overall prevalence of anti-HCV antibody of 0.43% among the general population aged 1–59 years [5], indicating that around 5.6 million people were infected with HCV. However, in addition to its status in the general Chinese population, the HCV prevalence is higher in specific populations, such as intravenous drug users, patients on maintenance hemodialysis, and paid blood donors.

Kuancheng County, Hebei Province lies in the North of China. Sodium benzoate and amphetamines were commonly used here 20 years ago, for recreational purposes and to alleviate fatigue. These drugs were often abused by groups of guests at private functions, with the majority of drug abusers being young farmers. We therefore conducted a pilot survey to analyze the virological and epidemiological characteristics of HCV in that region, and simultaneously measured serum hepatitis B antigen (HBsAg) and hepatitis B core antibody (anti-HBc) levels to assess the prevalence of HBV infection.

## Methods

### Study population

A total of 852 participants from 11 villages, randomly selected from Kuancheng County, Hebei Province, were included in the study. Participants were enrolled in the study regardless of age and sex, and all participants gave signed informed consent. The study protocol conformed to the ethical guidelines of the 1975 Declaration of Helsinki, was approved by the Ethics Committee at Peking University People’s Hospital, and was performed according to the guidelines of the International Conference on Harmonization for Good Clinical Practice.

### Data collection

A standardized questionnaire administered prospectively by hepatologists was used to record demographic data (including age and sex) and drug abuse. The data on drug abuse included the probable number and type (sodium benzoate or amphetamine) of drugs used, and the number of years from first drug use to December 2012. Serum anti-HCV, HBsAg, and anti-HBc were measured in all participants.

### Virological detection

Elecsys anti-HCV II assay (Roche Diagnostics GmbH, Penzberg, Germany) was used to detect anti-HCV [6] and the results were expressed as signal/cut-off (S/CO) ratios. The criteria for anti-HCV positivity and negativity were based on the manufacturer’s instructions. An S/CO ≥1.0 was considered positive. HCV RNA was measured in samples positive for anti-HCV using the COBAS AmpliPrep/COBAS TaqMan automated real-time polymerase chain reaction platform (Roche Molecular Systems, Pleasanton, CA, USA), with a lower limit of detection of 15 IU/mL and a lower limit of quantification of 43 IU/mL. Serum HBsAg and anti-HBc levels were detected using Elecsys HBsAg and anti-HBc assays (Roche Diagnostics), respectively, according to the manufacturer’s instructions, where an S/CO ratio ≥1.0 was positive for HBsAg and ≤1.0 was positive for anti-HBc. We defined past co-infection with HBV and HCV as seropositivity for both anti-HBc and anti-HCV, and current co-infection as seropositivity for both HBsAg and HCV RNA. Spontaneous clearance of HCV was defined as positive for anti-HCV but negative for HCV RNA in a patient without previous antiviral treatment.

### Statistical analysis

Continuous variables are presented as mean ± standard deviation or median, and compared using Student’s *t*-test, Kruskal–Wallis test, Mann–Whitney *U* test, or one-way analysis of variance. Categorical variables are presented as number and percentage, and compared using χ^2^ tests. The relationships between virological markers and injection times were analyzed by the trend χ^2^ test. Multiple logistic regression analysis was used to identify factors related to HCV infection or spontaneous clearance. All statistical analyses were performed using SPSS 16.0 (Chicago, IL, USA). Two-tailed *P* values < 0.05 were considered significant. When performing multiple comparisons among the four groups, the level of significance was adjusted to < 0.008.

## Results

### Study population and basic characteristics

**Figure 1:**
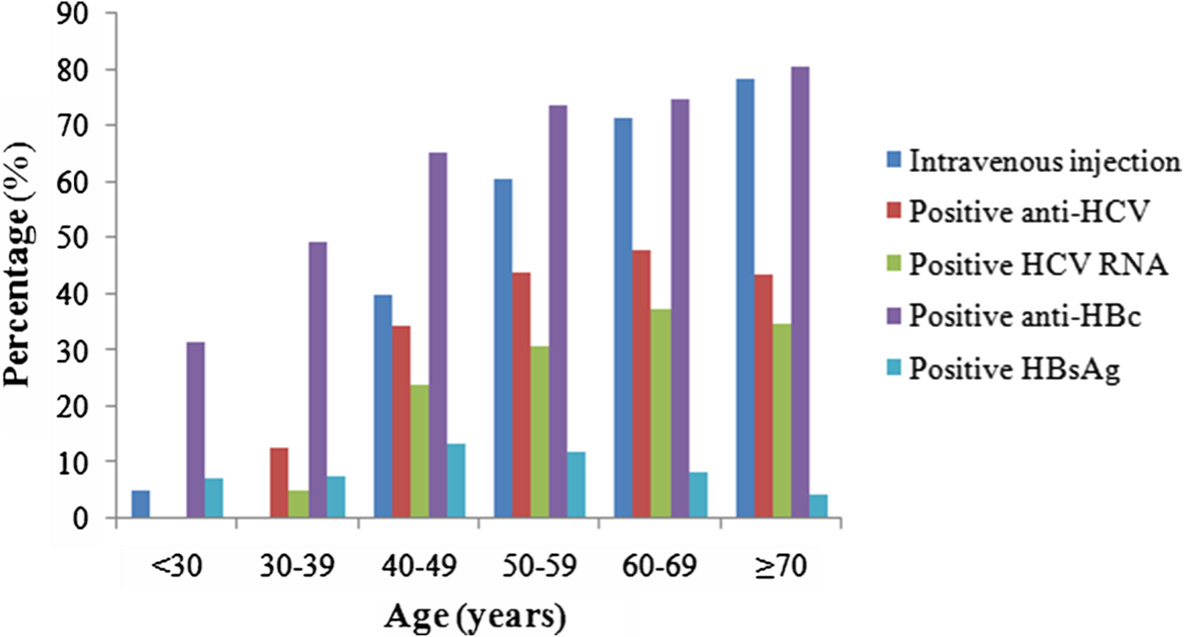
Percentages of intravenous injections and various positive HCV/HBV markers at different ages.

Among the 852 participants from 11 villages, 379 (44.5%) were men. The mean age was 45.16 ± 11.06 years (range: 12–86 years), with 41, 79, 235, 272, 179, and 46 participants aged <30, 30–39, 40–49, 50–59, 60–69, and ≥70 years, respectively. Four-hundred-twenty-five (49.9%) participants had used sodium benzoate or amphetamine at least once, administered by intravenous injection with needle sharing, and 147, 141, and 137 people had injected 1–10, 10–29, and ≥30 times, respectively. The number of injection times was related to the time from first injection to the survey (*r*_s_ = 0.911, *P* < 0.001). More men than women had injected intravenously (70.4% vs. 29.6%, χ^2^ = 229.801, *P* < 0.001), and people aged ≥40 years accounted for 99.5% of all participants (Figure 1). Compared with participants who had not injected intravenously, those who had injected were older, more likely to be male, and had a higher prevalence of anti-HCV, HCV RNA, and anti-HBc. However, there was no significant difference in the prevalence of HBsAg between the two groups (Table 1).

**Table 1:** **Characteristics of participants in relation to intravenous injection behavior**

**Factor**	**Injection**	**Non-injection**	**Statistics**	***P*** **value**
	***n*** **= 425**	***n*** **= 427**		
Age (years)	56.87 ± 9.23	45.96 ± 12.77	Z = −12.95	<0.001
Sex (male/female)	299/126	80/347	χ^2^ = 229.801	<0.001
Positive anti-HCV, *n* (%)	295 (69.4)	21 (4.9)	χ^2^ = 379.699	<0.001
Positive HCV RNA, *n* (%)	222 (52.2)	5 (1.2)	χ^2^ = 284.175	<0.001
Positive anti-HBc, *n* (%)	334 (78.6)	243 (56.9)	χ^2^ = 45.798	<0.001
Positive HBsAg, *n* (%)	44 (10.4)	47 (11.0)	χ^2^ = 0.096	0.757
Positive anti-HCV and anti-HBc, *n* (%)	242 (56.9)	14 (3.3)	χ^2^ = 291.818	<0.001
Positive HCV RNA and HBsAg, *n* (%)	12 (2.8)	0 (0)	χ^2^ = 12.229	<0.001

### HCV infection in an area of epidemic needle sharing

**Figure 2:**
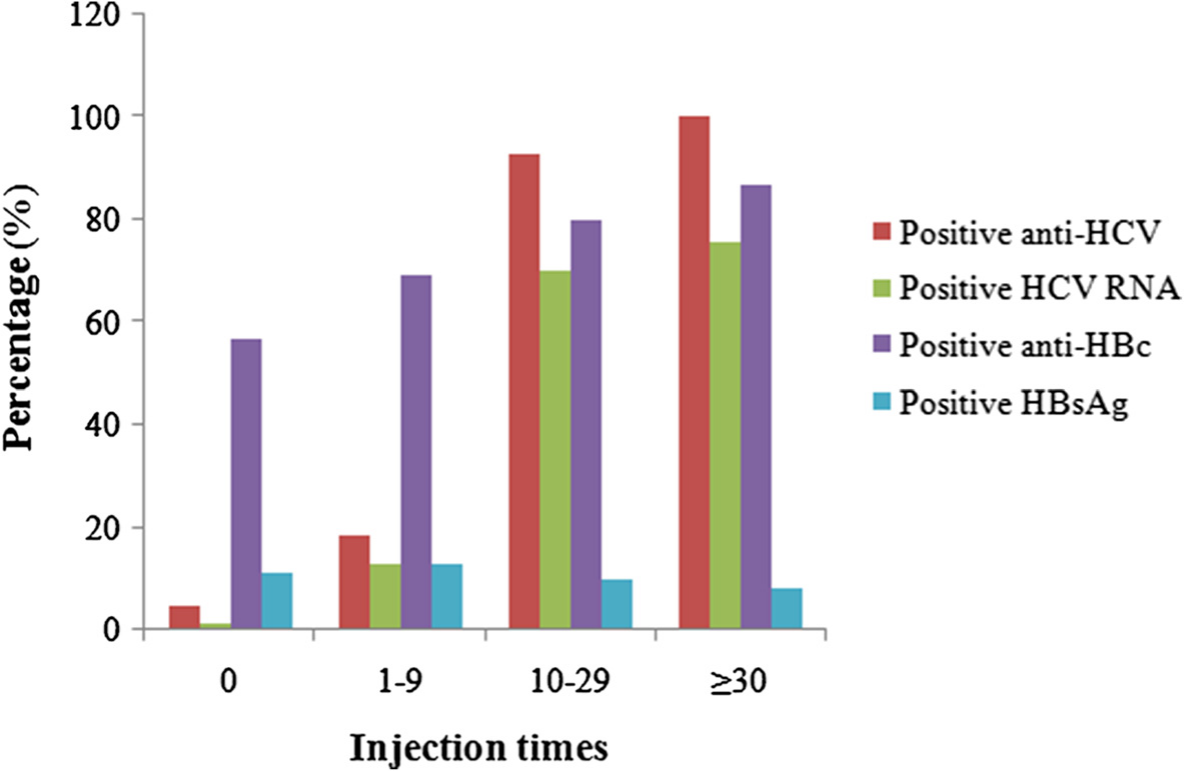
Percentages of various positive HCV/HBV markers at different injection times. (Trend tests: *P* < 0.001 between anti-HCV, HCV RNA, and anti-HBc and number of injections, respectively; *P* = 0.347 between HBsAg and injection times).

The overall prevalence of anti-HCV was 37.1% (316/852). An S/CO >10 was seen in 306 of 316 (96.8%) people who were positive for anti-HCV. HCV RNA was detected in 227 of 852 (26.6%) participants, and thus in 71.8% of those positive for anti-HCV (227/316). As shown in Table 2, serum anti-HCV status was related to sex, age, number of injection times, time from first injection, and anti-HBc positivity. Multiple regression analysis showed that anti-HCV positivity correlated significantly with male sex (odds ratio (OR) 1.218, 95% confidence interval (CI) 1.103–1.461, *P* < 0.001), older age (OR 1.922, 95% CI 1.898–1.947, *P* < 0.001) and more injections (OR 1.741, 95% CI 1.566–1.936, *P* < 0.001). The rate of spontaneous HCV RNA clearance was 28.2% (89/316), which was related to number of injections, time from first injection, and HBsAg positivity. Only HBsAg positivity correlated significantly with spontaneous HCV RNA clearance by multiple logistic regression analysis (OR 4.640, 95% CI 2.049–10.506, *P* < 0.001). HCV prevalence was significantly higher in people aged ≥40 years (306/732; 41.8%) compared with those <40 years (10/120; 8.3%) (χ^2^ = 49.498, *P* < 0.001) (Figure 1). Two-hundred-twenty-three of the 227 (98.2%) participants who were positive for HCV RNA were aged >40 years. The relationship between virological markers and number of injections is shown in Figure 2. Trend tests demonstrated that the prevalence of anti-HCV and HCV RNA were related to the number of injections (*P* < 0.001).

**Table 2:** **Factors related to serum anti-HCV status and spontaneous HCV clearance**

	**Anti-HCV**		***P*** **value**	**Spontaneous HCV clearance**		***P*** **value**
	**Positive (** ***n*** **= 316)**	**Negative (** ***n*** **= 536)**		**No (** ***n*** **= 227)**	**Yes (** ***n*** **= 89)**	
Sex (male/female)	194/122	185/351	<0.001	147/80	47/42	0.05
Age (years)	55.41 ± 9.41	49.04 ± 13.36	<0.001	56.18 ± 9.16	53.44 ± 9.82	0.20
No. of injections (median)	25	0	<0.001	25	20	0.003
Time from first injection (years, median)	26	0	<0.001	27	24	0.001
Positive anti-HBc (*n*)	256	321	<0.001	182	74	0.545
Positive HBsAg (*n*)	28	63	0.187	12	16	<0.001

### HBV infection in an area of epidemic needle sharing

The overall prevalence of anti-HBc and HBsAg was 67.7% (577/852) and 10.7% (91/852), respectively. HBsAg was detected in 87/577 (15.1%) of those who were positive for anti-HBc. Serum anti-HBc status was related to sex, age, number of injections, and duration from first injection. HBsAg was not related to the above parameters, but was related to anti-HBc S/CO ratio (Table 3). The prevalence of anti-HBc and HBsAg according to the number of injections are shown in Figure 2. Trend tests demonstrated that the prevalence of anti-HBc was related to the number of injections (*P* < 0.001), while the prevalence of HBsAg was not (*P* = 0.347).

**Table 3:** **Factors related to serum anti-HBc and HBsAg status**

	**Anti-HBc**		***P*** **value**	**HBsAg**		***P*** **value**
	**Positive (** ***n*** **= 577)**	**Negative (** ***n =*** **275)**		**Positive (** ***n*** **= 91)**	**Negative (** ***n =*** **761)**	
Sex (male/female)	277/300	102/173	0.003	45/46	334/427	0.313
Age (years)	53.31 ± 11.05	47.40 ± 14.12	<0.001	50.34 ± 9.88	51.53 ± 12.70	0.39
No. of injections (median)	5	0	<0.001	0	1	0.376
Time from first injection (years, median)	22	0	<0.001	0	19	0.473
Anti-HBc S/CO ratio				0.006	0.096	<0.001

### HBV/HCV co-infection in an area of epidemic needle sharing

The participants were divided into four groups: anti-HCV and anti-HBc positive (group 1); anti-HCV positive (group 2); anti-HBc positive (group 3); and anti-HCV and anti-HBc negative (group 4). A total of 30.0% of participants were group 1. Compared with participants who were positive for anti-HCV alone (group 2), those with HBV/HCV co-infection (group 1) had a lower anti-HBc S/CO ratio (*P* < 0.001), but sex, age, number of injections, and time from first injection were similar in both groups. In contrast, there was no significant difference in anti-HBc S/CO ratio between those in group 1 and those positive for anti-HBc alone (group 3). All five factors differed significantly between groups 1 and 4 (Table 4). The four groups were divided on the basis of HCV RNA and HBsAg. Comparing group 1 with the other groups, there were significant differences in the number of injections and time from first injection between groups 1 and 3, and in the number of injections, time from first injection to present, and anti-HBc S/CO ratio between groups 1 and 4 (Table 5).

**Table 4:** **Factors related to HCV and HBV infections assessed by anti-HCV and anti-HBV**

	**Anti-HCV and anti-HBc**				**Statistics**	***P***	**Multiple comparisons**		
	**Both positive (1)**	**Positive anti-HCV (2)**	**Positive anti-HBc (3)**	**Both negative (4)**			**1&2**	**1&3**	**1&4**
	**(** ***n*** **= 256)**	**(** ***n*** **= 60)**	**(** ***n*** **= 321)**	**(** ***n*** **= 215)**					
Sex (male/female)	159/97	35/25	118/203	67/148	χ^2^ = 60.067	<0.001	0.589	<0.001	<0.001
Age (years)	56.00 ± 9.20	52.87 ± 9.95	51.15 ± 11.91	45.88 ± 14.74	F = 28.732	<0.001	0.157	<0.001	<0.001
No. of injections (median)	25	20	0	0	χ^2^ = 554.047	<0.001	0.033	<0.001	<0.001
Time from first injection (years, median)	26	25.5	0	0	χ^2^ = 315.472	<0.001	0.441	<0.001	<0.001
Anti-HBc S/CO ratio	0.006	1.63	0.006	1.80	χ^2^ = 589.908	<0.001	< 0.001	0.319	<0.001

**Table 5:** **Factors related to HCV and HBV infections assessed by HCV RNA and HBsAg**

	**HCV RNA and HBsAg**				**Statistics**	***P***	**Multiple comparisons**		
	**Both positive (1)**	**Positive HCV RNA (2)**	**Positive HBsAg (3)**	**Both negative (4)**			**1&2**	**1&3**	**1&4**
	**(** ***n*** **= 12)**	**(** ***n*** **= 215)**	**(** ***n*** **= 79)**	**(** ***n*** **= 546)**					
Sex (male/female)	9/3	138/77	36/43	196/350	χ^2^ = 54.658	<0.001	0.547	0.057	0.011
Age (years)	57.17 ± 7.88	56.13 ± 9.23	49.30 ± 9.78	49.72 ± 13.41	F = 16.129	<0.001	0.998	0.036	0.042
No. of injections (median)	27.5	25	0	0	χ^2^ = 410.937	<0.001	0.902	<0.001	<0.001
Time from first injection (years, median)	29.5	27	0	0	χ^2^ = 247.583	<0.001	0.515	<0.001	<0.001
Anti-HBc S/CO ratio	0.006	0.10	0.006	0.28	χ^2^ = 105.000	<0.001	0.014	0.923	<0.001

## Discussion

National surveys in 1992 and 2006 showed a decline in the prevalence of anti-HCV from 3.2% to 0.43% in the general population in China, mainly as a result of the stringent administration and screening of blood donors and blood products. However, about 45% of HCV-infected people were in high-risk groups, such as intravenous drug users, patients undergoing maintenance hemodialysis, and paid blood donors [7]. The pooled prevalence of anti-HCV among intravenous drug users in China was 61.4% [8]. Mother-to-child transmission is a major cause of HBV infection in China, and other high-risk groups include hemodialysis patients, HIV-positive patients, and unprotected sexual exposure [9]. However, the impact of multiple injections on HBV infection, and the characterization of HCV and HBV co-infection following intravenous injection, are unclear.

The current survey found overall prevalence of anti-HCV and HCV RNA of 37.1% and 26.6%, respectively, which were higher than in the general population. Most people who were positive for anti-HCV were men older than 40 years. More injections and a longer duration from first injection were associated with a higher prevalence of anti-HCV and HCV RNA, and decreased spontaneous clearance of HCV. These data suggest that intravenous injection was the most important transmission route of HCV in Kuancheng County.

Family clustering of HBV infection occurs in China as a result of mother-to-child transmission. The rate of progression from acute to chronic HBV infection is related to age at infection, being as high as 90% for perinatally acquired infections but around 5% for adults [10]. The weighted prevalence of HBsAg and anti-HBc in the Chinese population aged 20–59 years was 8.97% and 43.9%, respectively [11]. The equivalent prevalence in the current study was 10.7% and 67.7%, respectively, and HBsAg was detected in 15.1% of those who were positive for anti-HBc, suggesting that most of them acquired HBV infection during adulthood. Anti-HBc, rather than HBsAg status, was related to sex, age, number of injections, and time from first injection, further suggesting that most people had acquired HBV infection during adulthood as a result of intravenous injection.

HCV/HBV co-infection is an important issue. The prevalence of both anti-HCV and anti-HBc positivity in the current study was 30.0%, while that of both HCV RNA and HBsAg positivity was 1.4%. Moreover, spontaneous clearance of HCV was associated with HBsAg (not anti-HBc), in agreement with previous reports [12,13]. Serum HBsAg levels are considered to be a surrogate marker of viral activity in infected hepatocytes, and in part represent the interaction between HBV and the host immune response. However, the role of HBsAg in HCV clearance is unclear, but may be influenced by some aspects of the viral life cycle or may involve the activation of innate immunity by HBsAg [13,14].

There were some limitations to the present study. It was not conducted by random cluster sampling. Furthermore, we had no data on occult HBV infection, defined as the presence of HBV DNA in the serum or liver of patients with negative HBsAg test results. Despite these limitations, the survey demonstrated a high prevalence of HCV and HBV infections (especially HCV infection) in areas where needle sharing was common, such as Kuancheng County. In contrast to the epidemiology of HBV infection, HCV infection in mainland China shows no family aggregation, but does demonstrate aggregations in regional, ethnic, or specific populations associated with particular life habits or religious customs. Regional aggregation is more pronounced in rural areas and populations, which have a higher prevalence of anti-HCV than in the general population. Surveys of HCV epidemiology should thus take these factors into account. Although populations at high risk of HCV infection have been paid increasing attention in clinical practice since 1993, no effective screening has been performed in high-risk areas. In the United States, around 75% of patients with HCV infection were born between 1945 and 1965, with a peak prevalence of 4.3% [15,16], and the US Preventive Services Task Force recommends offering one-time screening for HCV infection to this cohort [17]. In the present study, we found that injection of sodium benzoate largely occurred between the 1970s and 1990s, and 98.2% of patients positive for HCV RNA were over 40 years old. In contrast, the 2006 national survey showed no significant difference in the prevalence of HCV infection among different age groups in the general population, though the highest prevalence of 0.83% was found in persons aged 50–55 years [5]. However, we suggest that one-time screening for HCV infection should be offered to people in high-risk age groups in high-risk areas, and routine screening should be performed in high-risk populations.

## Conclusion

Prevalence of HCV, HBV and HCV/HBV co-infection is likely to be high in areas of China where needle sharing has been a common behavior, with intravenous injection being an important causative factor for chronic viral hepatitis, especially hepatitis C. More attention should be paid to routine screening for HCV and HBV in high-risk areas.

## References

[1] World Health Organization. Hepatitis C. (Updated July 2013) http://www.who.int/mediacentre/factsheets/fs164/en/index.html.

[2] World Health Organization. Hepatitis B. (Updated July 2013) http://www.who.int/mediacentre/factsheets/fs204/en/index.html.

[3] El-Serag HB (2012). Epidemiology of viral hepatitis and hepatocellular carcinoma. Gastroenterology.

[4] Xia GL, Liu CB, Cao HL, Bi SL, Zhan MY, Su CA (1996). Prevalence of hepatitis B and C virus infections in the general Chinese population: results from a nationwide cross-sectional seroepidemiologic study of hepatitis A, B, C, D, and E virus infections in China, 1992. Int Hepatol Commun.

[5] Chen YS, Li L, Cui FQ, Xing WG, Wang L, Jia ZY (2011). A sero-epidemiological study on hepatitis C in China. Chin J Epidemiol.

[6] Yang R, Guan W, Wang Q, Liu Y, Wei L (2013). Performance evaluation and comparison of the newly developed Elecsys anti-HCV II assay with other widely used assays. Clin Chim Acta.

[7] Gao X, Cui Q, Shi X, Su J, Peng Z, Chen X (2011). Prevalence and trend of hepatitis C virus infection among blood donors in Chinese mainland: a systematic review and meta-analysis. BMC Infect Dis.

[8] Xia X, Luo J, Bai J, Yu R (2008). Epidemiology of hepatitis C virus infection among injection drug users in China: systematic review and meta-analysis. Public Health.

[9] Cui Y, Jia J (2013). Update on epidemiology of hepatitis B and C in China. J Gastroenterol Hepatol.

[10] Boesecke C, Wasmuth JC, Mauss S, Berg T, Rockstroh J, Sarrazin C, Wedemeyer H (2013). Hepatitis B. Hepatology 2013: a clinical textbook.

[11] Liang XF, Bi SL, Yang WZ, Wang L, Cui G, Cui F (2009). Epidemiological serosurvey of hepatitis B in China - declining HBV prevalence due to hepatitis B vaccination. Vaccine.

[12] Dai CY, Huang JF, Hsieh MY, Lee LP, Ho CK, Chuang WL (2007). The role of gender on clearance of hepatitis C virus: a different story in an area endemic for hepatitis B and C. Gut.

[13] Yu ML, Dai CY, Huang CF, Lee JJ, Yeh ML, Yeh SM (2014). High hepatitis B virus surface antigen levels and favorable interleukin 28B genotype predict spontaneous hepatitis C virus clearance in uremic patients. J Hepatol.

[14] Eyre NS, Phillips RJ, Bowden S, Yip E, Dewar B, Locarnini SA (2009). Hepatitis B virus and hepatitis C virus interaction in Huh-7 cells. J Hepatol.

[15] Chou R, Cottrell EB, Wasson N, Rahman B, Guise JM (2012). Screening for hepatitis C virus infection in adults. Comparative Effectiveness Review no. 69. AHRQ publication no. 12-EHC090-EF.

[16] Smith BD, Patel N, Beckett GA, Jewett A, Ward JW (2011). Hepatitis C virus antibody prevalence, correlates and predictors among persons born from 1945 through 1965, United States, 1999–2008. Hepatology.

[17] Moyer VA, U.S. Preventive Services Task Force (2013). Screening for hepatitis C virus infection in adults: U.S. Preventive Services Task Force recommendation statement. Ann Intern Med.

